# Olfactory Stimulation Modulates Visual Perception Without Training

**DOI:** 10.3389/fnins.2021.642584

**Published:** 2021-08-02

**Authors:** Yoshiaki Tsushima, Yurie Nishino, Hiroshi Ando

**Affiliations:** ^1^National Institute of Information and Communications Technology, Center for Information and Neural Networks, Osaka, Japan; ^2^National Institute of Information and Communications Technology, Universal Communication Research Institute, Kyoto, Japan

**Keywords:** crossmodal perception, olfaction, motion perception, fMRI, psychophysics

## Abstract

Considerable research shows that olfactory stimulations affect other modalities in high-level cognitive functions such as emotion. However, little known fact is that olfaction modulates low-level perception of other sensory modalities. Although some studies showed that olfaction had influenced on the other low-level perception, all of them required specific experiences like perceptual training. To test the possibility that olfaction modulates low-level perception without training, we conducted a series of psychophysical and neuroimaging experiments. From the results of a visual task in which participants reported the speed of moving dots, we found that participants perceived the slower motions with a lemon smell and the faster motions with a vanilla smell, without any specific training. In functional magnetic resonance imaging (fMRI) studies, brain activities in the visual cortices [V1 and human middle temporal area (hMT)] changed based on the type of olfactory stimulation. Our findings provide us with the first direct evidence that olfaction modulates low-level visual perception without training, thereby indicating that olfactory-visual effect is not an acquired behavior but an innate behavior. The present results show us with a new crossmodal effect between olfaction and vision, and bring a unique opportunity to reconsider some fundamental roles of olfactory function.

## Introduction

Odors are powerful stimuli that not only evoke a sense of smell but also influence our mental states. In the real world, since we empirically know the effects of odors, we use them in our daily lives (e.g., aromatherapy). In scientific research fields, a number of studies on human crossmodal effects have shown that olfactory information interacts with other modalities in such high-level cognitive functions as emotions (Yoshida, 1979; Toller, 1988), memories (Rasch et al., 2007; Tamura et al., 2015; Dahmani et al., 2018), and social interactions (Baron, 1980; Dematte et al., 2007; Cook et al., 2015). In fact, it is often considered natural that olfactory stimulation influences such high-level cognitive functions, because the brain regions of olfaction and other cognitive functions are anatomically located near/overlap and are functionally associated (Frasnelli et al., 2010; Smitka et al., 2012). For example, Frasnelli et al. showed a correlation between olfactory performance and cortical structure at insular that is generally linked to emotion (Craig, 2009). On the other hand, it is relatively unknown that that olfactory stimulation affects low-level perception of different sensory modalities such as the detection and the identification of the spatial and temporal properties of sensory information (Groen et al., 2017). For example, Zhou et al. (2010) reported the case in which olfaction affected binocular rivalry (Lansing, 1964): the smell of rose/marker pen influenced the suppression time of binocular rivalry for the visual image of rose/marker pen. Also, Kuang and Zhang (2014) found that olfaction induced the distinctive motion perception with the specific training that participants repeatedly viewed the specific motion paired with the particular smell (e.g., banana smell with rightward motion). Although binocular rivalry or motion perception are phenomena of low-level perception, they involve the specific experiences, that is to say, the congruent combination of olfactory and visual experience of rose/marker pen, or the specific perceptual training. Does olfactory stimulation modulate other low-level perception without training? To test that possibility, we investigated the effect of olfactory stimulation on visual motion perception, which is known to involve various crossmodal interactions (Sekuler et al., 1997; Kitagawa and Ichihara, 2002; Soto-Faraco et al., 2003; Fujisaki and Nishida, 2009; Kuang and Zhang, 2014), thorough psychophysical and neuroimaging experiments.

## Materials and Methods (Psychophysical Experiments)

### Participants

Fourteen participants with normal or corrected vision and normal olfaction [seven females and seven males; mean age = 33.28 (SD = 6.74; ranging from 20 to 39)] participated in a series of experiments (“normal” in this study indicated a person who had never got vision/smell disorder for their daily lives, by their self-enumeration and recruiting agency’s reports). The *post hoc* analysis using PANGEA indicated that these sample size and experimental design (see below) yielded 80% power to detect *d* = 0.45 (Richard et al., 2003; Westfall, 2016).

### Visual Stimuli and Aroma (Lemon and Vanilla)

The visual stimuli were presented using Psychtoolbox 3 on Windows 7. Motion dots were presented within an annulus with an inner diameter of 1 degree and an outer diameter of 25 degrees. Each white dot was 0.2 degrees square with 800 dots in a frame. All the motion dots, which were randomly assigned to each location at the beginning of a trial to move out of the presented circle in a radial way. We controlled the life time of the motion dots based on the results of preliminary experiments to avoid a situation where the participant might learn the change of the dot density at the edge of the circle along with different motion dot speeds.

As the olfactory stimulations, we used lemon and vanilla smells for the following reasons: they were supposedly unrelated to any feature of motion perception; they were oppositely located at the sensory map of the odor descriptions in the previous studies (Edwards, 2007; Zarzo and Stanton, 2009). The lemon odor consisted of 67.26% limonene, 13.21% β-pinenne, 8.98% γ-terpinenne, and 2.11% citral. The vanilla odor consisted of 2.13% vanillin, 0.15% *p*-hydroxybenzaldehydel, 0.17% vanillic acid, 0.04% *p*-hydroxybenzonic acid, and 90.00% mono propylene glycol.

### Projecting System of Aroma Shooter

**FIGURE 1:**
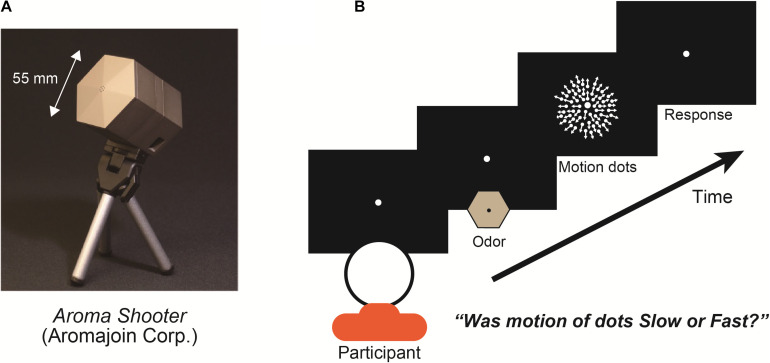
**(A)** Aroma projector machine, “Aroma Shooter.” **(B)** Experimental paradigm: participant fixated on display’s center with/without an odor for 1.0 s viewed expanding-motion dots for 1.0 s and reported their subjective motion speed as either fast or slow. Between each trial, at least a 4.0-s interval was set to avoid olfactory adaptation.

In the experiments, we used an aroma projector (Aroma Shooter, Figure 1A) that controlled the delivery of discrete pulses of odor to a participant’s nose with suitable temporal precision (Kim and Ando, 2010). Aroma Shooter has six aroma cartridge spaces. We put two lemon cartridges, two vanilla cartridges, and two odor-free cartridges in it. In the experiment, two cartridges were used in one trial to control the five kinds of olfactory stimulations in the whole experiment: full-lemon, half-lemon, full-vanilla, half-vanilla, and odor-free. Each odor was tested with regards to the motion dots; there was no simultaneous exposure to two different kinds of scents. In the full-lemon/vanilla condition, the Aroma Shooter used two lemon/vanilla cartridges. In half-lemon/vanilla condition, it used one lemon/vanilla cartridge and one odor-free cartridge. In the odor-free condition, it used two odor-free cartridges. With this method, participants perceived five kinds of olfactory stimulations with identical air strength.

### Experimental Designs and Analyses

At the beginning of each trial, the participants fixated on the display center during exposure to one of three olfactory stimulations (lemon, vanilla, or odor-free) for 1.0 s and viewed expanding-motion dots for 1.0 s (Figure 1B). Participants were asked to report the subjective motion speed of the dots as fast or slow and were instructed to give one of these two answers even when they had difficulty making a decision (a forced-choice paradigm). To examine the degree of the effect of the olfactory stimulations, we set two different levels of chemical density for the lemon and vanilla projectors: full and half. We set five types of olfactory stimulations: full-lemon, half-lemon, full-vanilla, half-vanilla, and odor-free. There were seven different dot motion speeds: 3.0, 3.5, 4.0, 4.5, 5.0, 5.5, and 6.0 degrees/s. Combinations of olfactory stimulation and dot motion were presented in a randomized and counterbalanced order for each participant. Before the experiments, we did not give the participants any information about the olfactory stimulations (especially what odor).

In the entire visual experiment, the total number of trials was 525: 5 olfactory stimulations × 7 speed motion dots × 15 repetitions. To reduce the olfactory adaptation and ventilate the experiment room, we set at least 4.0-s intervals between trials and separated the experiment into five sessions (105 trials each). The presentation order of these conditions was randomly determined. To evaluate their performance in motion speed perception in each olfactory stimulation, we calculated the point of subjective equality (PSE) of the motion speed in each olfactory stimulation condition [50% response point of fast and slow; Tanaka (1961)] and compared it to that in the odor-free condition (each PSE divided by PSE of odor-free condition).

To confirm how participants perceived the olfactory stimulations during the main visual experiment, we conducted the olfactory experiment after the main visual experiment. This second experiment was identical to the main visual experiment except that participants were asked to identify the smell: lemon, odor-free, or vanilla. In the olfactory experiment, the total number of trials was 105: 5 olfactory stimulations × 7 speed motion dots × 3 repetitions. To assess their performance, we measured the accuracy of the olfactory tasks; 33.3% correct was the chance-level because of three alternative choices.

## Results (Psychophysical Experiments)

**FIGURE 2:**
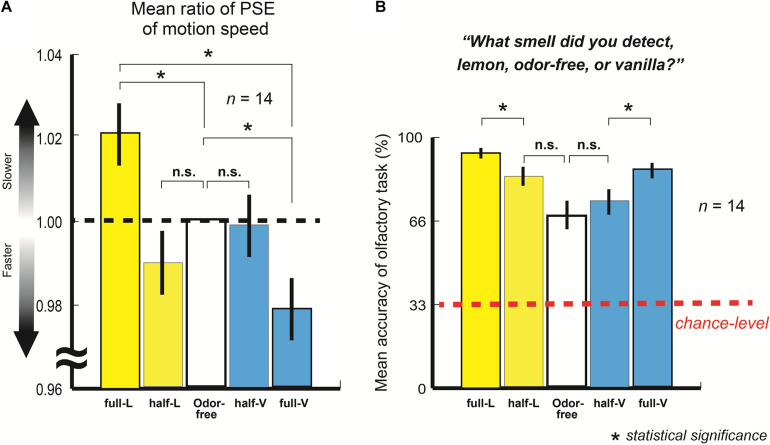
**(A)** Mean ratio of point of subjective equality (PSE) of motion speed with full-lemon, half-lemon, half-vanilla, and full-vanilla to PSE of odor-free. Error bars show standard errors. Participants perceived slower moving dots with lemon than with odor-free or vanilla even when stimuli were presented at identical motion speed. **(B)** Mean accuracy of olfactory task for full-lemon, half-lemon, odor-free, half-vanilla, and full-vanilla. Error bars show standard errors. Red dashed line represents chance-level accuracy (33.3% here).

Figure 2A represents the mean ratio of the PSE of the motion speed with full-lemon, half-lemon, half-vanilla, and full-vanilla to the PSE of the odor-free (*n* = 14; 525 trials each).

The results showed that the participants perceived the fastest motion with full-vanilla and the slowest motion with full-lemon, even when the stimuli were presented at the same motion speed (*t*_(13)_ = 6.19, *p* < 0.001, and *r* = 0.87). The findings clearly suggest that participants perceived a higher speed of motion dots in this order: vanilla > odor-free > lemon (full-lemon and odor-free: *t*_(13)_ = 2.85, *p* = 0.007, and *r* = 0.62: odor-free and full-vanilla: *t*_(13)_ = 6.19, *p* = 0.01, and *r* = 0.59). According to a brief questionnaire after the experiment, no participants were aware of exactly what odors were released in the experiments, although some did notice the existence of several kinds of odor. In addition, the participants did not mention that their performances were based on some kind of rule, e.g., they perceived faster motions with vanilla.

Figure 2B shows the mean accuracy of the olfactory task (*n* = 14; 105 trials each, also see section “Materials and Methods”). The results revealed that participants distinguished among three olfactory stimulations (all accuracies exceeded the chance level). This indicates that they adequately perceived each olfactory stimulation during the main visual experiment. They also perceived the half- and full-lemon/vanilla in different degrees of sensation because the mean accuracies of full-lemon/vanilla were significantly higher than those of half-lemon/vanilla (full-lemon and half-lemon: *t*_(13)_ = 4.23, *p* < 0.001, and *r* = 0.76: full-vanilla and half-vanilla: *t*_(13)_ = 3.61, *p* = 0.002, and *r* = 0.71). These results provide solid explanations about the task performance differences between the half- and full-lemon/vanilla in the main visual experiment.

To examine how this olfactory-visual effect occurred in the neural levels of the perceptual processing, we measured the functional magnetic resonance imaging (fMRI) activities in the visual areas while the participants performed the visual task. Note that participants were not people who joined the first experiments.

## Materials and Methods (fMRI Experiments)

### Participants

Twelve participants with normal or corrected vision and normal olfaction [nine females and three males; mean age = 21.05 (SD = 6.55; ranging from 20 to 39)] participated in a series of experiments. The *post hoc* analysis using PANGEA indicated that these sample sizes and experimental designs (see the below) yielded 80% power to detect *d* = 0.45 (Richard et al., 2003; Westfall, 2016).

### Visual Stimuli and Aroma

Stimuli and aroma were identical to the first psychophysical experiments.

### Projecting System of an MRI-Compatible Olfactory Stimulator

**FIGURE 3:**
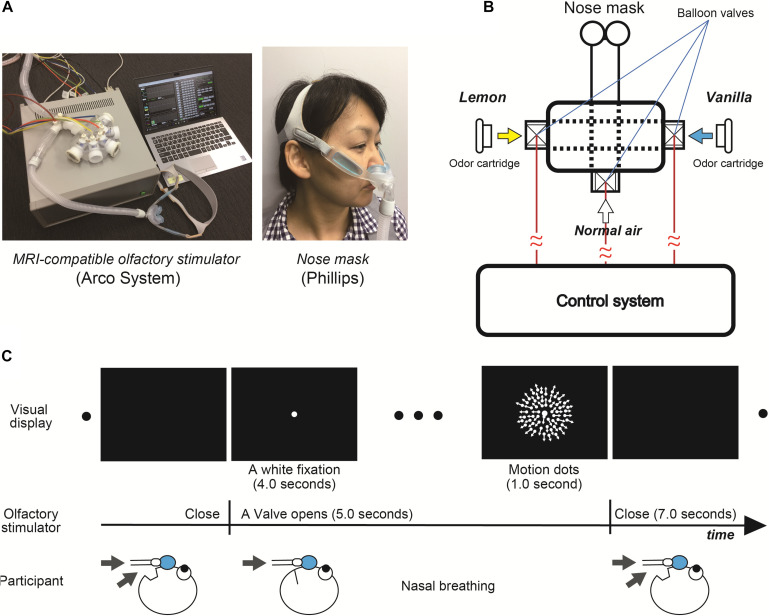
**(A)** Magnetic resonance imaging -compatible olfactory stimulator (Arco System) and nose mask (Phillips). **(B)** Schematic diagram of olfactory stimulator. Each balloon valve was opened and shut in experimental order. **(C)** Experimental paradigm of functional magnetic resonance imaging (fMRI) experiment.

We used an MRI-compatible olfactory stimulator (Arco System, Figures 3A,B). Unlike the first psychophysical experiments with Aroma Shooter (Figure 1A), since perception of olfactory stimulation depended on nasal respiration due to the mechanical system, they were asked to breathe through their nose as much as possible when white fixation and motion dots were presented (Figure 3C). Between each trial, at least a 7.0-s interval was set to avoid olfactory adaptation.

### Experimental Design

We conducted the identical visual tasks to the first psychophysical experiment except for three different speeds of motion dots, 3.0, 4.5, and 6.0 degrees/s and only one level of chemical density for the lemon and vanilla odors. To maximize the effect of olfactory stimulation, participants were asked to breathe through their nose as much as possible when the valve was opened (Figure 3C). Over the entire experiment, the total number of trials was 240: 120 trials with odor-free, 60 with lemon, and 60 with vanilla stimulus. Each olfactory condition was accompanied by three different-motion-speed dots. To reduce the olfactory adaptation, we set at least a 7.0-s interval between trials, separated the experiment into eight sessions (30 trials each), and set the number of odor-free conditions as twice that of the other conditions. The presentation order of these conditions was randomized across conditions.

### Functional Magnetic Resonance Imaging Data Acquisition and Analyses

Magnetic Resonance Imaging data were obtained by a 3T MRI scanner (Magnetom Trio; Siemens, Erlangen, Germany) using a 12-channel head coil at the Center for Information and Neural Networks (Osaka, Japan). To achieve a high spatial resolution whole-brain fMRI with a standard TR, we used multi-band echo planar imaging (EPI) implemented by CMRR^1^. An interleaved T2^∗^-weighted gradient-echo planar imaging scan acquired functional images that covered the entire brain (TR, 2,000 ms; TE, 30 ms; flip angle, 80°; multi-band acceleration factor = 3, partial Fourier = 6/8; voxel size, 2 × 2 × 2 mm; and number of slices, 75). We also collected anatomical images (T1-weighted, MP-RAGE) whose imaging parameters were as follows: TR, 1,900 ms; TE, 2.48 ms, flip angle, 9°; and voxel size, 1 × 1 × 1 mm.

Image pre-processing and statistical analyses were run by SPM12 (Wellcome Trust Centre for Neuroimaging, UCL, SPM). The acquired fMRI data underwent slice-timing correction and motion correction by SPM12. The data were then co-registered to the within-session high-resolution anatomical images of the same slices used for EPI and subsequently to the whole-head high-resolution anatomical images. Then the images were spatially normalized to the MNI template, their voxel size was resampled as 2 × 2 × 2 mm voxels. Finally, they were smoothed; the FWHM was 8 mm. Anatomical labels for the region of interest analysis were defined using the SPM anatomy toolbox (SPM Anatomy Toolbox) and WFU_PickAtlas.

### Calculation of Ratio of Response Time in fMRI Experiment

Since, we found relatively large individual difference of response time in fMRI experiment, we calculated the ratio of response time (Response time at 3.0, 4.5, or 6.0 degrees/s divided by individual averaged response time at all dot speed). The unconverted data is available in Supplementary Figure 5.

## Results (fMRI Experiments)

**FIGURE 4:**
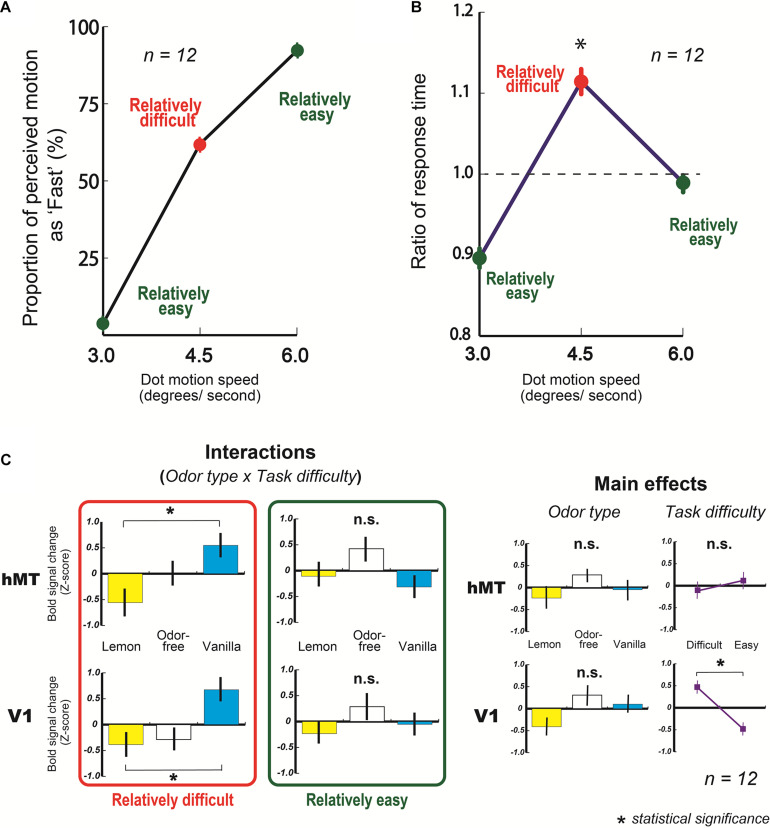
Results of fMRI experiments. **(A)** Mean behavioral response of fast (%) as a function of dot speed (degrees/s). **(B)** Mean ratio of response time in fMRI experiment as a function of dot speed (degrees/s). A dashed line represents averaged ratio of response time at all dots speed condition (1.0 here, also see section “Materials and Methods”). The ratio of response time at 4.5 degrees/s condition was significantly higher than at 3.0 and 6.0 degrees/s. **(C)** Averaged blood oxygen level-dependent (BOLD) signal changes (Z-score) in V1 and hMT with three types of odor, lemon, odor-free, and vanilla. Error bars show standard errors.

From the behavioral results, we found that the mean proportions of motion perceived as fast at 3.0 and 6.0 degrees/s were 3.8 ± 1.3 and 92.2 ± 1.9%, respectively, (Figure 4A, green circles on graph). Also, the mean response of fast at 4.5 degrees/s was 62.0 ± 5.1% (Figure 4A, a red circle on the graph).

In addition, the mean response time for making a decision at 4.5 degrees/s was significantly longer than at 3.0 and 6.0 degrees/s (3.0 and 4.5 degrees/s: *t*_(11)_ = 4.19, *p* < 0.001, and *r* = 0.79: 4.5 degrees/s and 6.0 degrees/s: *t*_(__11__)_ = 2.60, *p* = 0.01, and *r* = 0.62) (Figure 4B). These results suggest that participants had more difficulty making a decision (fast or slow) with 4.5 degree/s motion dots than at 3.0 (slowest motion) or 6.0 degrees/s (fastest motion).

From the fMRI results, the amount of activity in the human middle temporal area (hMT), which principally reflects the processed motion signals (Mikami et al., 1986; Newsome and Pare, 1988; Rees et al., 2000; Wall et al., 2008; Meier et al., 2018), did not change based on the type of olfactory stimulation at the slowest and fastest motion speeds (relatively easy) (Figure 4C, an upper graph in the green rectangle, also see upper graphs in Supplementary Figure 1); however, they changed significantly with the type of olfactory stimulation at 4.5 degrees/s of motion (relatively difficult) (Figure 4C, an upper graphs in the red rectangle). The following are the two-way ANOVA results: odor type x task difficulty: *F*_(2, 22)_ = 4.84, *p* = 0.018, and *partial*η^2^ = 0.31. In addition, the hMT activity amount with vanilla was significantly higher than with lemon at the relatively difficult condition (*t*_(11)_ = 2.79, *p* = 0.009, and *r* = 0.64) (Figure 4C, an upper graph in the red rectangle). These results indicate that the fMRI activities in the visual cortex (hMT) changed significantly based on the type of olfactory stimulation when participants had more difficulty making a decision on the visual task. Similar activities were observed in the primary visual area (V1), which is the first stage of the cortical processing of visual information. The following are the results of a two-way ANOVA: odor type x task difficulty: *F*_(2, 22)_ = 3.64, *p* = 0.043, and *partial*η^2^ = 0.25 (Figure 4C, bottom graphs, also see bottom graphs in Supplementary Figure 1), and the amount of V1 activity with vanilla was significantly higher than that with lemon at the relatively difficult condition (*t*_(11)_ = 3.50, *p* = 0.002, and *r* = 0.73) (Figure 4C, a bottom graph in the red rectangle). In ANOVA analyses of V1 and hMT, the main effects of odor type and task difficulty were not significant except the task difficulty in V1 (Figure 4C).

## Discussion

Our results demonstrated three significant points in the effect of olfactory stimulations on visual perception. First, the behavioral results certainly revealed that olfactory stimulation changed the visual perception without any specific training such as viewing the fast-motion with vanilla smell or slow-motion with lemon smell. This is the first direct evidence of olfactory-visual effect at the low-level of perceptual processing without training. This finding indicates that olfactory-visual effect is not an acquired but an innate behavior, because the obtained crossmodal phenomenon between olfaction and vision did not require any specific training. Confirming the presence of innate olfactory-visual effect gives us an opportunity to uncover not only crossmodal interaction with olfactory stimulations but also the roots in the biological evolution of olfactory system, because it might provide us with a clue to an underlying role of olfactory function such as olfaction for survival navigation (Jacobs, 2012; Dahmani et al., 2018).

Second, the fMRI results showed that the olfactory stimulations directly or indirectly affected the brain activity in the visual cortices. In particular, the brain activity in the visual cortices (V1 and hMT) significantly changed based on the type of olfactory stimulation when participants had relative difficulty making decisions in the visual task (Figure 4C, graphs in red rectangle). To put it plainly, our perceptual system does not integrate the stimulus information for a task (motion dots here) with the information of the other modality (lemon and vanilla odors here) when the task stimulus satisfies certain criteria for making a decision; however, it does integrate the stimulus information for a task with the information of other modality when it fails to meet certain criteria. This is in accord with the general view of crossmodal effect (Shams et al., 2000; Ernst and Banks, 2002). Furthermore, since the fMRI results in V1 resembled those for hMT, this phenomenon appears to happen at an earlier perceptual level [also, the fMRI data in the other visual areas (V2 and V3) are available in Supplementary Figure 2]. This result is consistent with our view from the behavioral findings, that is to say, this implies the existence of crossmodal interactions between olfaction and vision at the low-level of perceptual processing (Jadauji et al., 2012). By the same token, olfactory-visual interaction is presumably built in “hard-wiring” of the nervous system as an innate behavior (Mayer, 1963; Herrnstein, 1972). One might assume that the olfactory stimulations affected not motion perception but just participants’ arousal level, which caused the different sense of visual perception (Cano et al., 2006; Lee et al., 2014; Kim et al., 2017). To check that possibility, we measured fMRI activities in the thalamus that are associated with the level of arousal (Portas et al., 1998; Cano et al., 2006). The results showed that those activities were not related to the type of olfactory stimulations in this study (Supplementary Figure 3). Hence, we concluded that the change of arousal level by an olfactory stimulation did not mainly contribute to our current findings. Moreover, we analyzed fMRI activities in the olfactory cortex for getting more insights of this olfactory-visual effect, but we could not find the significant BOLD signal differences with the olfactory stimulations used in this study (Supplementary Figure 4). To see the clearer relationship between olfactory stimulations and fMRI activities in the olfactory cortex, we might need to control the valence of chemical substances more severely (Anderson et al., 2003).

Third, our findings raise a new question. Why do the participants perceive slower movement with the lemon smell and faster movement with the vanilla smell? Perhaps the captured attention might have induced different speeds of moving dots, because an attended object was perceived to move faster than an unattended one (Turatto et al., 2006). For example, they could have paid less attention to the moving dots and perceived slower-moving dots, because the lemon odor might exploit more attentional resources than the vanilla odor. However, this is not likely. If that was the case, the fMRI activities in V1 and hMT, which are crucially influenced by attentional state (O’Craven et al., 1997; Somers et al., 1999; Tsushima et al., 2006), changed with the type of the olfactory stimulation. But, they did not change with those odors used in this study (see in the main effects in Figure 4C). These results imply that this possibility cannot directly explain the current findings. Another possible explanation is that the lemon and vanilla odors might have influenced our participants’ perception of time. A number of psychological and neural studies have focused on our ability to keep and perceive our internal clocks (Allman et al., 2014), and recent investigations have indicated that internal clocks are modulated by colors (Thönes et al., 2018) and odors (Yue et al., 2016). In our study, the lemon odor might have increased the speed of the internal clock, which might in turn have created a sense of slowness for the visual inputs at the perceptual level, and vice versa with the vanilla odor (Yamamoto and Miura, 2016). From another perspective, the current finding might be related to a fast-lemon issue; people usually link a lemon with being fast rather than slow (Woods et al., 2013). This suggests that some symbolic elements of lemon (or vanilla) correlate with speed (fast or slow). However, the fast-lemon issue is mostly argued by semantic or synaesthetic association in high-level cognitive processing (Brown, 1958; Martino and Marks, 1999; Spence, 2011; Chen and Spence, 2017). In addition, we identified a slow-lemon effect because the lemon odor made participants perceive slower-moving dots. Therefore, the present findings might not directly contribute to the interpretation of the fast-lemon issue. Although use of lemon and vanilla seemed to be effective for observation of this kind of crossmodal phenomenon (Hanson-Vaux et al., 2013), one of the limitations of this study is caused by the fact that we tested only these two odors. In order to get a better understanding of this phenomenon, a broader range of odors should be tested in the future study. Although further studies are needed to fully understand the psychological/neurological mechanism of obtained results, our findings encourage us to re-examine not only other crossmodal interactions induced by olfaction but also some fundamental roles of the olfactory functions.

## Data Availability Statement

The original contributions presented in the study are included in the article/Supplementary Material, further inquiries can be directed to the corresponding author.

## Ethics Statement

The studies involving human participants were reviewed and approved by the Ethics Committee of the National Institute of Information and Communications Technology, Japan. The participants provided their written informed consent to participate in this study. Written informed consent was obtained from the individual(s) for the publication of any potentially identifiable images or data included in this article.

## Author Contributions

YT and YN conceived the research idea, designed the experiment, and conducted the experiments. All authors discussed the results and commented on the manuscript.

## Conflict of Interest

The authors declare that the research was conducted in the absence of any commercial or financial relationships that could be construed as a potential conflict of interest. The reviewer GV declared a past collaboration with one of the authors YT to the handling editor.

## Publisher’s Note

All claims expressed in this article are solely those of the authors and do not necessarily represent those of their affiliated organizations, or those of the publisher, the editors and the reviewers. Any product that may be evaluated in this article, or claim that may be made by its manufacturer, is not guaranteed or endorsed by the publisher.

## Footnotes

1
https://www.cmrr.umn.edu/multiband/

## References

[B1] Allman M. J. Teki S. Griffiths T. D. Meck W. H. (2014). Properties of the internal clock: first-and second-order principles of subjective time. *Annu. Rev. Psychol.* 65 743–771. 10.1146/annurev-psych-010213-115117 24050187

[B2] Anderson A. K. Christoff K. Stappen I. Panitz D. Ghahremani D. G. Glover G. (2003). Dissociated neural representations of intensity and valence in human olfaction. *Nat. Neurosci.* 6 196–202. 10.1038/nn1001 12536208

[B3] Baron R. A. (1980). Olfaction and human social behavior: effects of pleasant scents on physical aggression. *Basic Appl. Soc. Psychol.* 1 163–172. 10.1207/s15324834basp0102_5 33486653

[B4] Brown R. W. (1958). Is a boulder sweet or sour? *Contemp. Psychol.* 3 113–115. 10.1037/005792

[B5] Cano M. Bezdudnaya T. Swadlow H. A. Alonso J.-M. (2006). Brain state and contrast sensitivity in the awake visual thalamus. *Nat. Neurosci.* 9 1240–1242. 10.1038/nn1760 16964255

[B6] Chen Y. Spence C. (2017). Assessing the role of the ‘Unity Assumption’ on multisensory integration: a review. *Front. Psychol.* 8:445. 10.3389/fpsyg.2017.00445 PMC5374162 28408890

[B7] Cook S. Fallon N. Wright H. Thomas A. Giesbrecht T. Field M. (2015). Pleasant and unpleasant odors influence hedonic evaluations of human faces: an event-related potential study. *Front. Hum. Neurosci.* 9:661. 10.3389/fnhum.2015.00661 PMC4681274 26733843

[B8] Craig A. D. (2009). How do you feel – now? The anterior insula and human awareness. *Nat. Rev. Neurosci.* 10 59–70. 10.1038/nrn2555 19096369

[B9] Dahmani L. Patel R. M. Yang Y. Chakravarty M. M. Fellows L. K. Bohbot V. D. (2018). An intrinsic association between olfactory identification and spatial memory in human. *Nat. Commun.* 9:4162. 10.1038/s41467-018-06569-4 PMC6191417 30327469

[B10] Dematte M. L. Österbauer R. Spence C. (2007). Olfactory cues modulate facial attractiveness. *Chem. Senses* 32 603–610. 10.1093/chemse/bjm030 17507456

[B11] Edwards M. (2007). *Fragrances of the World 2008: Parfums du Monde*, 24th Edn. Sydney: Fragrances of the World.

[B12] Ernst M. O. Banks M. S. (2002). Humans integrate visual and haptic information in a statistically optimal fashion. *Nature* 415 429–433. 10.1038/415429a 11807554

[B13] Frasnelli J. Lundström J. N. Boyle J. A. Djordjevic J. Zatorre R. J. Jones-Gotman M. (2010). Neuroanatomical correlates of olfactory performance. *Exp. Brain Res.* 201 1–11. 10.1007/s00221-009-1999-7 19730837

[B14] Fujisaki W. Nishida S. (2009). Audio-tactile superiority over visuo-tactile and audio-visual combinations in the temporal resolution of synchrony perception. *Exp. Brain Res.* 198 245–259. 10.1007/s00221-009-1870-x 19499212

[B15] Groen I. I. A. Silson E. H. Baker C. I. (2017). Contributions of low- and high-level properties to neural processing of visual scenes in the human brain. *Phil. Trans. R. Soc. B* 372:20160102. 10.1098/rstb.2016.0102 28044013 PMC5206270

[B16] Hanson-Vaux G. Crisinel A. S. Spence C. (2013). Smelling shapes: crossmodal correspondences between odors and shapes. *Chem. Senses* 38 161–166. 10.1093/chemse/bjs087 23118203

[B17] Herrnstein R. J. (1972). Nature as nurture: behaviorism and the instinct doctrine. *Behaviorism* 1 23–52.

[B18] Jacobs L. F. (2012). From chemotaxis to the cognitive map: the function of olfaction. *Proc. Natl. Acad. Sci. U.S.A.* 109 10693–10700. 10.1073/pnas.1201880109 22723365 PMC3386877

[B19] Jadauji J. B. Djordjevic J. Lundström J. N. Pack C. C. (2012). Modulation of olfactory perception by visual cortex stimulation. *J. Neurosci.* 32 3095–3100. 10.1523/jneurosci.6022-11.2012 22378882 PMC6622023

[B20] Kim D. Ando H. (2010). “Development of directional olfactory display,” in *Proceedings of the 9th ACM SIGGRAPH International Conference on Virtual Reality Continuum and Its Applications in Industry (VRCAI), Proceedings of the 9th ACM SIGGRAPH Conference on VRCAI 2010, Seoul, South Korea, December 12-13, 2010*, Seoul, 143–144.

[B21] Kim D. Lokey S. Ling S. (2017). Elevated arousal levels enhance contrast-perception. *J. Vision* 17:14. 10.1167/17.2.14 28245495

[B22] Kitagawa M. Ichihara S. (2002). Hearing visual motion in depth. *Nature* 415 429–433. 11894093 10.1038/416172a

[B23] Kuang S. Zhang T. (2014). Smelling directions: olfaction modulates ambiguous visual motion perception. *Sci. Rep.* 4:5796. 10.1038/srep05796 PMC4107342 25052162

[B24] Lansing R. W. (1964). Electroencephalographic correlates of binocular rivalry in man. *Science* 146 1325–1327. 10.1126/science.146.3649.1325 14207465

[B25] Lee T.-H. Baek J. Lu Z.-L. Mather M. (2014). How arousal modulates the visual contrast sensitivity function. *Emotion* 14 978–984. 10.1037/a0037047 24932842 PMC4172527

[B26] Martino G. Marks L. E. (1999). Perceptual and linguistic interactions in speeded classification: tests of the semantic coding hypothesis. *Perception* 28 903–923. 10.1068/p2866 10664781

[B27] Mayer E. (1963). *Animal Species and Evolution.* Cambridge, MA: Harvard University Press.

[B28] Meier K. Partanen M. Giaschi D. (2018). Neural correlates of speed-tuned motion perception in healthy adults. *Perception* 47 660–683. 10.1177/0301006618771463 29683390

[B29] Mikami A. Newsome W. T. Wurtz R. H. (1986). Motion selectivity in macaque visual cortex. II. Spatiotemporal range of directional interactions in MT and V1. *J. Neurophysiol.* 55 1328–1339. 10.1152/jn.1986.55.6.1328 3734858

[B30] Newsome W. T. Pare E. B. (1988). A selective impairment of motion perception following lesions of the middle temporal visual area (MT). *J. Neurosci.* 8 2201–2211. 10.1523/jneurosci.08-06-02201.1988 3385495 PMC6569328

[B31] O’Craven K. M. Rosen B. R. Kwong K. K. Treisman A. Savoy R. L. (1997). Voluntary attention modulates fMRI activity in human MT-MST. *Neuron* 18 591–598. 10.1016/s0896-6273(00)80300-1 9136768

[B32] Portas C. M. Rees G. Howseman A. M. Josephs O. Turner R. Frith C. D. (1998). A specific role for the thalamus in mediating the interaction of attention and arousal in humans. *J. Neurosci.* 18 8979–8989. 10.1523/jneurosci.18-21-08979.1998 9787003 PMC6793555

[B33] Rasch B. Büchel C. Gais S. Born J. (2007). Odor cues during slow-wave sleep prompt declarative memory consolidation. *Science* 315 1426–1429. 10.1126/science.1138581 17347444

[B34] Rees G. Friston K. Koch C. (2000). A direct quantitative relationship between the functional properties of human and macaque V5. *Nat. Neurosci.* 3 716–723. 10.1038/76673 10862705

[B35] Richard F. D. Bond C. F. Jr. Stokes-Zoota J. J. (2003). One hundred years of social psychology quantitatively described. *Rev. Gen. Psychol.* 7 331–363. 10.1037/1089-2680.7.4.331

[B36] Sekuler R. Sekuler A. B. Lau R. (1997). Sound alters visual motion perception. *Nature* 385:308. 10.1038/385308a0 9002513

[B37] Shams L. Kamitani Y. Shimojo S. (2000). What you see is what you hear. *Nature* 408 788–788. 10.1038/35048669 11130706

[B38] Smitka M. Puschmann S. Buschhueter D. Gerber J. C. Witt M. Honeycutt N. (2012). Is there a correlation between hippocampus and amygdala volume and olfactory function in healthy subjects? *Neuroimage* 59 1052–1057. 10.1016/j.neuroimage.2011.09.024 21967725

[B39] Somers D. C. Dale A. M. Seiffert A. E. Tootell R. B. H. (1999). Functional MRI reveals spatially attentional modulation in human primary visual cortex. *Proc. Natl. Acad. Sci. U.S.A.* 96 1663–1668. 10.1073/pnas.96.4.1663 9990081 PMC15552

[B40] Soto-Faraco S. Kingstone A. Spence C. (2003). Multisensory contributions to the perception of motion. *Neuropsychologia* 41 1847–1862. 10.1016/s0028-3932(03)00185-4 14527547

[B41] Spence C. (2011). Crossmodal correspondences: a tutorial review. *Atten. Percept. Psychophys.* 73 971–995. 10.3758/s13414-010-0073-7 21264748

[B42] Tamura K. Hamakawa M. Okamoto T. (2015). Olfactory modulation of colour working memory: how does citrus-like smell influence the memory of orange colour? *PLoS One* 13:e0203876. 10.1371/journal.pone.0203876 30212534 PMC6136778

[B43] Tanaka Y. (1961). Determination of the point of subjective equality in the constant method procedure. *Jpn. Psychol. Res.* 3 193–199. 10.4992/psycholres1954.3.193

[B44] Thönes S. von Castell C. Iflinger J. Oberfeld D. (2018). Color and time perception: evidence for temporal overestimation of blue stimuli. *Sci. Rep.* 8:1688. 10.1038/s41598-018-19892-z PMC5786107 29374198

[B45] Toller S. V. (1988). Odours, emotion and psychophysiology. *Int. J. Cosmetic Sci.* 10 171–197. 10.1111/j.1467-2494.1988.tb00016.x 19456922

[B46] Tsushima Y. Sasaki Y. Watanabe T. (2006). Greater disruption due to failure of inhibitory control on an ambiguous distractor. *Science* 314 1786–1788. 10.1126/science.1133197 17170308

[B47] Turatto M. Vescovi M. Valsecchi M. (2006). Attention makes moving objects be perceived to move faster. *Vis. Res.* 47 166–178. 10.1016/j.visres.2006.10.002 17116314

[B48] Wall M. B. Lingnau A. Ashida H. Smith A. T. (2008). Selective visual responses to expansion and rotation in the human MT complex revealed by functional magnetic resonance imaging adaptation. *Eur. J. Neurosci.* 27 2747–2757. 10.1111/j.1460-9568.2008.06249.x 18547254

[B49] Westfall J. (2016). *PANGEA: Power Analysis for General Anova Design. Unpublished Manuscript.* Available online at: http://jakewestfall.org/publications/pangea.pdf (accessed October 11, 2016).

[B50] Woods A. T. Spence C. Butcher N. Deroy O. (2013). Fast lemons and sour boulders: testing crossmodal correspondences using an internet-based testing methodology. *i-Perception* 4 365–379. 10.1068/i0586 24349696 PMC3859554

[B51] Yamamoto K. Miura K. (2016). Effect of motion coherence on time perception relates to perceived speed. *Vis. Res.* 123 56–62. 10.1016/j.visres.2015.11.004 26721584

[B52] Yoshida M. (1979). “Descriptive and emotional profiles of odours and their preferences,” in *Preference Behaviour and Chemoreception*, ed. Kroeze J. H. A. (London: Informational Retrievel Limited), 83–92.

[B53] Yue Z. Gao T. Chen L. Wu J. (2016). Odors bias time perception in visual and auditory modalities. *Front. Psychol.* 7:535. 10.3389/fpsyg.2016.00535 PMC4841154 27148143

[B54] Zarzo M. Stanton D. T. (2009). Understanding the underlying dimensions in perfumers’ odor perception space as a basis for developing meaningful odor maps. *Atten. Percept. Psychophys.* 71 225–247. 10.3758/app.71.2.225 19304614

[B55] Zhou W. Jiang Y. He S. Chen D. (2010). Olfaction modulates visual perception in binocular rivalry. *Curr. Biol.* 20 1356–1358. 10.1016/j.cub.2010.05.059 20598540 PMC4226334

