# Supplementary material for: Olfactory Stimulation Modulates Visual Perception Without Training
Source: Front Neurosci. 2021 Aug 2;15:642584. doi: 10.3389/fnins.2021.642584 (PMC8364961; doi:10.3389/fnins.2021.642584)
Supplement: Supplementary file 1 [file Data_Sheet_1.docx]

**Supplementary Material**

**“Olfactory stimulation modulates visual perception without training.”**

Yoshiaki Tsushima, Yurie Nishino, and Hiroshi Ando

**BOLD signal changes (Z-score) in hMT and V1 at 3.0 and 6.0 degrees/ second (relatively easy)**

**
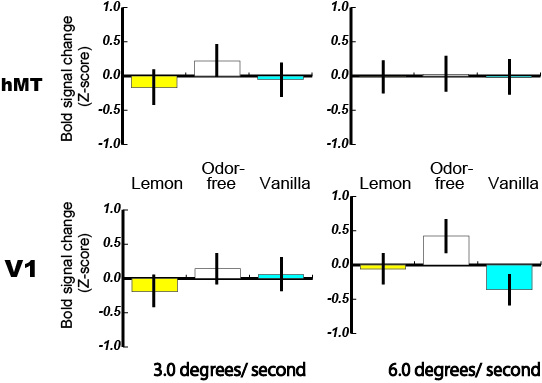
**

**Figure 1|** Averaged BOLD signal changes (Z-score) in hMT and V1 at 3.0 and 6.0 degrees/ second (relatively easy) with three types of odor, lemon, odor-free, and vanilla. Error bars show standard errors.

At all condition in each area, there were no significant differences of fMRI activities between the type of olfactory stimulation. This indicates that opposite results of BOLD signal changes between 3.0 and 6.0 degrees/ second (e.g. lemon > odor-free > vanilla and lemon < odor-free < vanilla) did not result in no significant differences (“cancel out”) of fMRI activities between the type of olfactory stimulation (Figure. 4C graphs in the green rectangle).

**BOLD signal changes (Z-score) in V2 and V3**

**Figure 2 |** Results of fMRI experiments. Averaged BOLD signal changes (Z-score) in V2 and V3 with three types of odor. Error bars show standard errors.

The results of two-way ANOVA in V2 were as follows: odor type x task difficulty: *F* (2, 22) = 2.44, *p* = .11, partial η^2^ = .18. On top of that, the amount of V2 activity with vanilla was not significantly higher than with lemon (*t*(11) = 6.19, *p* =.03, *r* = .87). Also, the results of two-way ANOVA in V3 were as follows: odor type x task difficulty: *F* (2, 22) = 2.43, *p* = .11, partial η^2^ = .18. On top of that, the amount of V3 activity with vanilla was not significantly higher than with lemon, *p* = .20). The main effects of odor type and task difficulty were not significant.

Unlike V1 and hMT, we could not find statistically significant differences of BOLD signal changes between the type of olfactory stimulation even when relatively difficult conditions (4.5 degrees/ second) in V2 and V3 (There was a slightly similar tendency as hMT and V1 in V2: vanilla odor > lemon odor). But this might be reasonable, because the functional connectivity between hMT and V1 is stronger than other visual areas (Born and Bradley, 2005).

**BOLD signal changes (Z-score) in the thalamus**

**
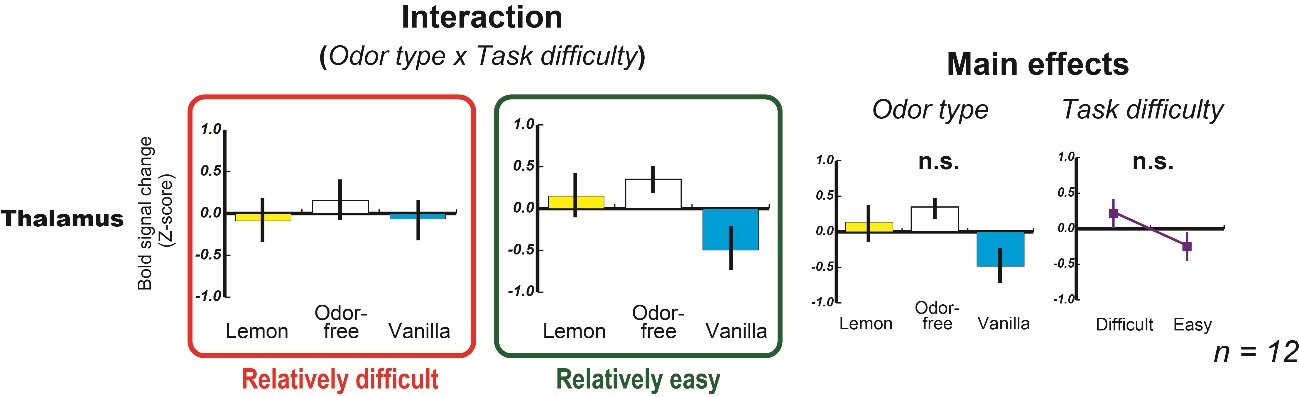
**

**Figure 3 |** Results of fMRI experiments. Averaged BOLD signal changes (Z-score) in thalamus with three types of odor. Error bars show standard errors.

The results of two-way ANOVA in thalamus were as follows: odor type x task difficulty: *F* (2, 22) = 1.68, *p* = .21, partial η^2^ = .13. The main effects of odor type and task difficulty were not significant.

Therefore, we concluded that the olfactory stimulations in this study did not significantly change participants’ arousal level.

**BOLD signal changes (Z-score) in the olfactory cortex by odors**

**Figure 4 |** Results of fMRI experiments. Averaged BOLD signal changes (Z-score) in the olfactory cortex with three types of odor. Error bars show standard errors.

The results of one-way ANOVA in the olfactory cortex was as follows: *F* (2, 22) = 2.36, *p* = .12. Unfortunately, we could not find the fMRI activity differences in the olfactory by the olfactory stimulations used in this study, lemon, odor-free, and vanilla. Maybe, it needs more severe control the valence of the chemical substance for finding the relationship between fMRI activities in the olfactory cortex and olfactory stimulations (Anderson et al. 2003).

**Reaction time in fMRI experiments.**

**Figure 5 |** Averaged response time for making a decision in fMRI experiment as a function of dot speed (Unconverted data as the ratio). Error bars show standard errors.

Since we found relatively large individual difference of response time in fMRI, we calculated the ratio of response time (Response time at 3.0, 4.5, or 6.0 degrees/ second divided by individual averaged response time at all dot speed.), and represented the converted data in the main text (Figure 4B).

**REFERENCES**

Born R, Bradley D. Structure and function of visual area MT. Annu Rev Neurosci **28**: 157-189. (2005).

Anderson, A.K. et al. Dissociated representations of intensity and valence in human olfaction*. Nat. Neurosci.* **3**, 196-202 (2003).
